# Workplace Exposures and the Risk of Amyotrophic Lateral Sclerosis

**DOI:** 10.1289/ehp.0900580

**Published:** 2009-05-11

**Authors:** Fang Fang, Patricia Quinlan, Weimin Ye, Marie K. Barber, David M. Umbach, Dale P. Sandler, Freya Kamel

**Affiliations:** 1 Epidemiology Branch, National Institute of Environmental Health Sciences, National Institutes of Health, Department of Health and Human Services, Research Triangle Park, North Carolina, USA; 2 Department of Medical Epidemiology and Biostatistics, Karolinska Institutet, Stockholm, Sweden; 3 Department of Medicine, Division of Occupational and Environmental Medicine, University of California, San Francisco, California, USA; 4 Westat, Durham, North Carolina, USA; 5 Biostatistics Branch, National Institute of Environmental Health Sciences, National Institutes of Health, Department of Health and Human Services, Research Triangle Park, North Carolina, USA

**Keywords:** amyotrophic lateral sclerosis, chemicals, relative risk, risk factors, workplace exposures

## Abstract

**Background:**

Occupation has been suggested to play a role in amyotrophic lateral sclerosis (ALS) etiology, but detailed information on the importance of specific workplace exposures is lacking.

**Objectives:**

Our aim was to assess the relationship between workplace exposures and the risk of ALS and to evaluate potential interactions between these exposures and smoking.

**Methods:**

We conducted a case–control study in New England between 1993 and 1996, comprising 109 cases and 253 controls who completed a structured interview covering occupations and workplace exposures. Unconditional logistic regression models were used to estimate the odds ratios (ORs) and 95% confidence intervals (CIs) for ALS. Analyses were conducted among the entire study population and after stratification by smoking.

**Results:**

We observed a higher risk of ALS for construction workers excluding supervisors (OR = 2.9; 95% CI, 1.2–7.2) and precision metal workers (OR = 3.5; 95% CI, 1.2–10.5). Self-reported exposures to paint strippers; cutting, cooling, or lubricating oils; antifreeze or coolants; mineral or white spirits; and dry cleaning agents each appeared to be associated with a 60–90% higher risk. Specific chemicals related to a > 50% increase in risk of ALS included aliphatic chlorinated hydrocarbons, glycols, glycol ethers, and hexane. Relative risks associated with these workplace exposures and chemicals were greater among nonsmokers and persisted in mutually adjusted models.

**Conclusions:**

Our data suggest that certain occupations and workplace exposures may be associated with increased risk of ALS. These results need to be confirmed in independent populations.

Amyotrophic lateral sclerosis (ALS) is a devastating neurodegenerative disease of the motor neurons with largely unknown etiology. Various workplace exposures have been implicated in ALS development, although few of these relationships have been confirmed (Armon 2001; Mitchell 2000; Nelson 1995). For example, some studies found that farmers and agriculture workers were at increased risk of ALS (Granieri et al. 1988; Gunnarsson et al. 1991; McGuire et al. 1997), but others did not (Sutedja et al. 2007; Weisskopf et al. 2005a). Inconsistent results also have been noted for the relationship between ALS and military service (Schulte et al. 1996; Sutedja et al. 2007; Weisskopf et al. 2005a, 2005b).

We conducted a case–control study in New England in 1993–1996 to assess the associations between workplace exposures and ALS, based on self-reported lifetime occupational history and information on exposure to specific chemical and physical agents. Because an association between formaldehyde and ALS has recently been reported (Weisskopf et al. 2009), we assessed this exposure separately. We also examined whether the associations between workplace exposures and ALS differed between smokers and nonsmokers, because smoking has been suggested as an independent risk factor for ALS (Fang et al. 2006; Kamel et al. 1999; Nelson et al. 2000; Sutedja et al. 2007; Weisskopf et al. 2004) and because exposure to chemicals found in cigarette smoke might influence the metabolism or activity of workplace chemicals.

## Materials and Methods

### Patients and controls

Our case–control study has been described in detail elsewhere (Kamel et al. 2002). One of the primary aims of the study was to assess the associations between occupational exposures, including lead, and the risk of ALS. All study participants (cases and controls) were recruited between 1993 and 1996. Sequential ALS cases were recruited from two major referral centers in New England. Board-certified neurologists specializing in motor neuron diseases made diagnoses of ALS based on World Federation of Neurology El Escorial criteria (Brooks 1994). Cases were required to live in New England for at least 50% of the year, to be mentally competent, and to speak English. In total, 71% of eligible cases participated in the study (*n* = 111). About 85% of the cases were enrolled within 1 year after diagnosis and the remainder within 2 years.

Population controls who met the same eligibility criteria as cases and had not been diagnosed with a neurodegenerative disease by a physician were identified through random telephone screening. Controls were frequency-matched to cases on sex, age (30–55, 56–65, and 66–80 years), and region within New England (Boston metropolitan area, eastern Massachusetts, and rest of New England). A total of 354 eligible controls were contacted, and 270 (76%) were enrolled in the study, including 256 that completed the entire questionnaire.

All study participants provided written informed consent. The study proposal was approved by the institutional review boards of the National Institute of Environmental Health Sciences, Tufts–New England Medical Center, and Brigham and Women’s Hospital.

### Exposure assessment

A structured interview completed within 1 month of enrollment collected information on demographics, lifestyle, and occupational history. Study participants reported all jobs held for at least 2 years since they were 19 years of age, with information on industry name, occupational activity, calendar years when a job was initiated and terminated, and the average working hours per week. Industries and occupations were coded using the 1990 Census Industrial and Occupational Classification Codes (U.S. Bureau of Labor Statistics 2008). We restricted the analyses to occupations held before ALS diagnosis among the cases and 2 years before the interview date among the controls (because all the cases were enrolled within 2 years of diagnosis). Two cases and three controls with missing occupational data for all relevant time periods were excluded, leaving 109 cases and 253 controls in the present analysis.

The association between military service (ever served in Army, Air Force, Navy, Marines, Coast Guard, or Armed Forces, branch not specified) and the risk of ALS was assessed separately. Relative risk variations by service branch, duration of service (1–2, 3–5, and > 5 years), and wartime service (1917–1918 World War I, 1942–1944 World War II, 1950–1953 Korean War, or 1965–1973 Vietnam War) were further investigated.

Participants reported separately exposure to any of 21 specific agents in response to the question, “On any of your jobs, were you exposed 10 times or more to . . . .” Those who responded positively were asked about total years exposed and average days of exposure per year. The latter two variables were multiplied to calculate lifetime days of exposure, and categorized as “1–399,” “400–1,999,” and “≥ 2,000” days, giving approximately equal numbers in each category for most exposures. Specific chemical exposures likely to result from these agents were determined by an industrial hygienist *a priori* [see Supplemental Material, Table 1, available online (doi:10.1289/ehp.0900580.S1 via http://dx.doi.org/)].

We identified occupations possibly involving formaldehyde exposure (Agency for Toxic Substances and Disease Registry 1999), and an industrial hygienist created an *a priori* three-level scale for probability of exposure in these occupations [0–1, 1, and 2; see Supplemental Material, Table 2 (doi:10.1289/ehp.0900580. S1)]. We calculated weighted hours of formaldehyde exposure from each job as the product of job duration (assuming 50 working weeks per year) and weekly working hours weighted by the three-level scale (weights of 0.5, 1, and 2 were given to levels 0–1, 1, and 2, respectively); these were then summed over all jobs to derive weighted lifetime hours of exposure. Finally, the lifetime hours of exposure were categorized into tertiles based on the distribution among the controls.

Interviewers estimated the overall quality of each interview at its conclusion. Of the 362 interviews, 264 were classified as high quality, 93 as generally reliable, and five as questionable.

### Statistical analysis

We used odds ratios (ORs) and their corresponding 95% confidence intervals (CIs) derived from unconditional logistic regression models to assess the relative risks of ALS. Individuals with more than one occupation were included in multiple categories. The matching variables age, sex, and area of residence were included in all models. Smoking history (ever or never) and educational level (≤ high school or > high school) were included as potential confounders.

We tested potential linear trends of ORs with the lifetime days of exposure to each workplace agent as well as exposure probability and weighted exposure duration of formaldehyde using a continuous variable with four values (0, 1, 2, and 3) representing unexposed and the three exposure categories. To assess potential interactions between smoking and the workplace exposures, we conducted analyses stratified by smoking (ever/never). We also evaluated the significance of the interactions by adding a term for the interaction of a specific exposure and smoking to the models. We report two-tailed *p*-values and consider any *p*-value < 0.05 as statistically significant. We used SAS version 9.1 (SAS Institute Inc., Cary, NC, USA) for all statistical analyses.

## Results

Characteristics of cases and controls are presented in Table 1. The median age at diagnosis for cases was 60 years (range, 30–79 years) and the median age at 2 years before interview for controls was 59 years (range, 29–78 years). Diagnosis was made 14.3 months after the onset of symptoms on average. Overall, 27 (25%) of the cases had a bulbar onset and 82 (75%) had a trunk or limb onset. Cases had a median of four different jobs (range, 1–10) before ALS diagnosis. Controls also had a median of four jobs (range, 1–13) up to 2 years before the date of interview (Table 1).

Construction trades and precision production were both associated with a higher risk of ALS (OR = 2.5; 95% CI, 1.0–5.8 and OR = 2.2; 95% CI, 1.1–4.4, respectively) (Table 2). Construction workers excluding supervisors had a 2.9-fold risk of ALS (95% CI, 1.2–7.2), whereas precision metalworking in particular was associated with a 3.5-fold risk of ALS (95% CI, 1.2–10.5). Transportation and material-moving workers had a nonsignificantly higher risk of ALS (OR = 1.9; 95% CI, 0.9–4.3); those using motor vehicles had an even higher risk (OR = 2.2; 95% CI, 0.9–5.6). Service-related occupations, excluding private household and protective service, were associated with a lower risk of ALS (OR = 0.3; 95% CI, 0.1–0.7). The occupational cate gory “farming, forestry, and fishing” was not associated with the risk of ALS (OR = 1.0; 95% CI, 0.2–4.2).

In total, 24 cases (19%) and 49 controls (22%) had served in the military. Military service overall was not associated with the risk of ALS (OR = 1.2; 95% CI, 0.7–2.4). No clear variation of the ORs was noted when military service was subgrouped by branch, duration, or wartime service (data not shown).

Table 3 shows the association between workplace exposures and ALS. We include for comparison previously reported data showing an association of ALS with lead exposure (Kamel et al. 2002). Exposure to paint strippers; cutting, cooling, or lubricating oils; antifreeze or coolants; mineral or white spirits; or dry cleaning agents was each associated with a 1.6- to 1.9-fold risk of ALS, although only the association with cutting, cooling, or lubricating oils achieved statistical significance (Table 3). Analyses stratified by smoking status showed that ORs were generally larger among non-smokers than smokers, especially for cutting, cooling, or lubricating oils; antifreeze and coolants; and mineral or white spirits (Table 3). In an additional model including all three exposures among the nonsmokers, the ORs were 3.9 for cutting, cooling, or lubricating oils; 1.9 for antifreeze and coolants; and 2.9 for mineral or white spirits; none was statistically significant (data not shown). Some interaction with smoking was suggested for antifreeze and coolants (*p-*value for interaction = 0.06) as well as cutting, cooling, or lubricating oils (*p*-value for interaction = 0.10); none of the other workplace exposures had a significant interaction with smoking (all *p-*values for interaction > 0.05).

No exposure except lead (Kamel et al. 2002) showed a clear-cut dose–response with lifetime days of exposure (Table 4). The trend for cutting, cooling, or lubricating oils was also significant (*p* = 0.04), but the dose–response curve was not monotonic. Other exposures had a similar nonmonotonic trend, with elevated ORs at medium but not high levels of exposure duration. We found substantial although imprecise elevations in the risk of ALS for 400- to 1,999-day exposure to cutting, cooling, or lubricating oils and for ≥ 2,000-day exposure to dyes or printing inks (Table 4).

Table 5 shows the association between ALS and chemicals inferred from the work-place exposures. Aliphatic chlorinated hydrocarbons, ethylene/propylene glycols, glycol ethers, heptane, and hexane were each associated with a 1.5- to 1.7-fold risk of ALS. Analyses stratified by smoking status showed generally larger ORs among nonsmokers than smokers (Table 5). The interaction of smoking with exposure was statistically significant for glycol ethers (*p* = 0.02) and xylene (*p* = 0.03) and was borderline significant for aliphatic chlorinated hydrocarbons (*p* = 0.06) and ethylene/propylene glycols (*p* = 0.06).

Because workers may have been exposed to several chemicals simultaneously, we examined associations of ALS with each chemical among the nonsmokers while adjusting for the five chemicals associated with ALS in the entire population (aliphatic chlorinated hydrocarbons, ethylene/propylene glycols, glycol ethers, heptane, and hexane; one per each model). Associations between ALS and most chemicals diminished in these analyses except for aliphatic chlorinated hydrocarbons, ethylene/propylene glycols, glycol ethers, and hexane (data not shown).

Exposure to formaldehyde was not associated with the risk of ALS overall (OR = 0.8; 95% CI, 0.5–1.5), and the ORs did not vary greatly by exposure probability (0–1, 1, and 2) or weighted exposure duration (≤ 10,000, 10,001–40,000, and > 40,000 hr) (Table 6). An additional analysis was conducted comparing individuals who had a weighted exposure to formaldehyde > 60,000 hr (four cases and four controls) to unexposed individuals, giving an OR of 3.0 (95% CI, 0.7–12.9).

In the interview, the subjects were asked two additional questions: “Did you usually clean your hands with solvents or thinners on any job?” and “Did you ever feel sick or high from an exposure at work?” In total, 78 subjects (26 cases and 52 controls) responded positively to the first question, giving an OR of 1.2 (95% CI, 0.7–2.2), and 57 subjects (16 cases and 41 controls) responded positively to the second question, giving an OR of 0.7 (95% CI, 0.3–1.3).

Because there may still be residual confounding by age in models where it was included as a categorical matching variable, we ran additional models further adjusted for age as a continuous variable. Estimates of the relative risks of ALS were substantially unchanged in these models (data not shown). Finally, we repeated all analyses presented above in three separate sets of sensitivity analyses after excluding *a*) five subjects whose interviews were characterized as questionable; *b*) seven cases who had reported a family history of ALS; or *c* ) 19 cases who had reported a previous trip to islands in the Western Pacific. These analyses did not give substantially changed conclusions (data not shown).

## Discussion

In the present study, we found that construction workers excluding supervisors and precision metalworkers were at a higher risk of ALS. These findings are consistent with earlier findings that heavy labor (Breland and Currier 1967; Chio 2000; Nelson 1995) and metal exposure (Kamel et al. 2002, 2005) may be related to ALS risk. We found no relationship of ALS risk either to military service or to the occupational group farming, fishing, or forestry. Some previous studies have suggested that farming was related to ALS (Granieri et al. 1988; Gunnarsson et al. 1991; McGuire et al. 1997), but other studies found no relationship (Sutedja et al. 2007; Weisskopf et al. 2005a). Earlier studies on the relationship of military service to ALS risk also had inconsistent findings—some suggested a positive association, but others did not (Schulte et al. 1996; Sutedja et al. 2007; Weisskopf et al. 2005a, 2005b).

Reasons for these inconsistencies are not yet clear. Different approaches for classification of occupations, lack of information on specific workplace exposures related to the occupations, and potential confounding or effect modification by other risk factors are all possible explanations. Using crude approximations of specific exposures—for example, military service—may account for some inconsistencies. That the association between military service and ALS may be time limited, as reported by another recent study (Horner et al. 2008), may also explain the different findings we have noted. “Farming” is also a general term not necessarily related to specific exposures.

Earlier studies have suggested that solvents or other chemicals are associated with a higher risk of ALS, albeit without detailed information on any specific solvent or chemical (Chancellor et al. 1993; Chio et al. 1991; Gunnarsson et al. 1992; Morahan and Pamphlett 2006). Other studies did not confirm these suggestions (Gait et al. 2003; McGuire et al. 1997). Our study extends previous work by considering specific solvents and chemicals. Workplace agents with potential relations to ALS in our data included paint strippers; cutting, cooling, or lubricating oils; antifreeze and coolants; mineral or white spirits; and dry cleaning agents. Specific chemicals responsible for these associations may include aliphatic chlorinated hydrocarbons, glycols, glycol ethers, and hexane. These associations with specific chemicals may also explain our findings for particular occupations. For example, precision metalworkers are likely exposed to metal particles and cutting, cooling, or lubricating oils. The fact that construction workers but not their supervisors had a higher risk provides further support for a role in ALS etiology of specific exposures that only the workers experienced.

Several lines of evidence suggest biological plausibility for the observed associations between workplace exposures and ALS. *n*-Hexane, an organic solvent associated with a higher risk of ALS in our study, is a classic neurotoxicant (Ritchie et al. 2001a) that caused polyneuropathy affecting both sensory and motor neurons (Chang 1990) and was associated with Parkinson’s disease (Canesi et al. 2003). Hydrocarbons were shown to induce persisting changes in protein expression and neurotransmitter levels in the central nervous system and to impair neurobehavioral function in rats at the “real-world” concentrations experienced by human beings (Ritchie et al. 2001b). Acute intoxication with glycol ethers increased free-radical production in the nervous system in rats (Kadiiska and Mason 2000), and an acute oral challenge with ethylene glycol in humans induced abnormal gait, loss of reflexes, central nervous system depression, and convulsions (Hess et al. 2004).

Our study did not find an overall association between exposure to formaldehyde and ALS risk as reported earlier (Weisskopf et al. 2009), although we noted an imprecise 3-fold risk of ALS among the most highly exposed group (> 60,000 weighted exposure hr). These results should be interpreted with caution, given the reliance of both studies on self-reported exposure as well as the small number of exposed cases in each study.

In evaluating associations between work-place exposures and diseases, it is important to consider other environmental factors such as smoking that might modify the neurotoxicity of the exposures. Although methodologic problems should be considered as a possible explanation for our findings, we found no consistent differences in covariates between smokers and nonsmokers, and there is no apparent reason why nonsmoking cases would be more likely than smoking cases to report workplace exposures. Beyond methodologic issues, biological explanations should also be considered. It is possible, for example, that chemical exposures from smoking are so great that the additional exposure to workplace chemicals becomes irrelevant. Another possible explanation is induction by smoking of metabolic enzymes. *n*-Hexane is hydroxylated at the 2 and 5 positions primarily by cytochrome P450 (CYP) 2E1, leading to formation of the neurotoxic metabolite 2,5-hexanedione (Zhang et al. 2006). Although smoking induces CYP2E1, suggesting that smokers should be more sensitive to neurotoxic effects of hexane, smokers may also have increased levels of other P450 isozymes (e.g., CYP1A1 and CYP1A2), leading to increased detoxification of *n*-hexane. In addition, constituents of cigarette smoke may compete with *n*-hexane for CYP2E1, forcing the latter into detoxifying pathways.

Our study has several strengths. First, ALS diagnoses were confirmed by board-certified neurologists using well-recognized diagnostic criteria. Second, we had detailed information on workplace exposures in addition to job and industry titles, independent inference of specific chemical exposures by an industrial hygienist, and information on important covariates such as smoking status. The limitations of the present study should also be appreciated. First, it is a case–control study with self-reported exposure. However, the presence of some associations (e.g., lead–ALS) but not others (e.g., mercury–ALS) mitigates to some extent concern about recall bias. Second, ALS cases were enrolled from tertiary care centers and might not be representative of cases in the population; for example, they might tend to live longer (Lee et al. 1995), so an association with an exposure that shortened survival might be missed. However, 85% of cases were enrolled within 1 year and the remainder within 2 years of diagnosis; survival bias should thus have minimal impact. Further, the median survival time between first diagnosis and death was 28 months (Kamel et al. 2008), comparable to survival time in other studies (Logroscino et al. 2008). Third, we did not find a monotonic increase of ORs with increasing duration of most exposures. This phenomenon is frequently seen in occupational studies and has several potential explanations (Stayner et al. 2003), including misclassification (i.e., highly exposed individuals underreporting exposure and vice versa), healthy worker effect (i.e., highly exposed individuals who developed health outcomes avoiding further contact with the agent), and true biological leveling off in the highly exposed group (i.e., the adverse health consequence of the exposures saturates at a specific level). Finally, statistical power to identify associations was low. However, for a disease as rare as ALS, the number of cases available for study is typically limiting, and few studies have sufficient statistical power to detect the moderate associations usually reported for occupational exposures. In the future, it may be possible to combine results from several studies to increase power.

## Conclusions

The present study suggests that ALS risk may be elevated in construction and precision metalworkers. Our results also suggest that workplace exposures—for example, to cutting, cooling, or lubricating oils—and specific chemicals, including aliphatic hydrocarbons, glycols, glycol ethers, and *n*-hexane, may be associated with a higher risk of ALS, especially among nonsmokers. Further studies with detailed industrial hygienist review of job task–based occupational histories are warranted to confirm these findings in other populations and to investigate the underlying mechanisms.

## Figures and Tables

**Table 1:** Basic characteristics of cases (*n* = 109) and controls (*n* = 253).

Characteristic	Cases [No. (%)]	Controls [No. (%)]
Age (years)		
30–55	38 (34.9)	83 (32.8)
56–65	37 (33.9)	73 (28.9)
66–80	34 (31.2)	97 (38.3)
Sex		
Male	66 (60.6)	156 (61.7)
Female	43 (39.4)	97 (38.3)
Region		
Boston metropolitan area	32 (29.4)	101 (39.9)
Eastern Massachusetts	28 (25.7)	53 (21.0)
New England	49 (44.9)	99 (39.1)
Education		
> High school	71 (65.1)	200 (79.0)
≤ High school	38 (34.9)	53 (21.0)
Smoking		
Never	32 (29.4)	105 (41.5)
Ever	77 (70.6)	148 (58.5)
Total jobs [median (range)]	4 (1–10)	4 (1–13)

**Table 2:** Occupations and the risk of ALS.^a^

Occupation	No. of controls (*n* = 253)	No. of cases (*n* = 109)	OR (95% CI)^b^
Executive, administrative, and managerial	71	25	0.9 (0.5–1.5)
Management related	36	6	0.4 (0.2–1.1)
Engineers, architects, and surveyors	92	37	1.1 (0.7–1.9)
Technician and related support	39	8	0.5 (0.2–1.1)
Sales	68	27	1.0 (0.6–1.7)
Administrative support, including clerical	80	42	1.4 (0.9–2.4)
Private household	8	2	0.5 (0.1–2.7)
Protective service	10	6	1.3 (0.4–3.8)
Service, excluding household or protective	43	8	0.3 (0.1–0.7)
Farming, forestry, and fishing	6	3	1.0 (0.2–4.2)
Mechanics and repairers	19	14	1.6 (0.7–3.4)
Construction trades	13	14	2.5 (1.0–5.8)
Precision production	21	19	2.2 (1.1–4.4)
Plant and system operators	1	0	—
Machine operators, assemblers, and inspectors	38	18	0.8 (0.4–1.6)
Transportation and material moving	16	14	1.9 (0.9–4.3)
Handlers, equipment cleaners, helpers, and laborers	21	8	0.7 (0.3–1.6)

a For analysis of each occupation, individuals who never had that occupation served as the reference group. Occupations were categorized using the 1990 Census Industrial and Occupational Classification Codes (U.S. Bureau of Labor Statistics 2008).

b Calculated from logistic regression models. All models were adjusted for age (< 56, 56–65, and > 65 years), sex, area of residence (Boston metropolitan area, eastern Massachusetts, and rest of New England), smoking, and educational level.

**Table 3:** Self-reported workplace exposures and the risk of ALS, overall and stratified by smoking.^a^

	Overall			Smokers			Nonsmokers		
Exposure	No. of controls (*n* = 253)	No. of cases (*n* = 109)	OR (95% CI)^b^	No. of controls (*n* = 148)	No. of cases (*n* =77)	OR (95% CI)^c^	No. of controls (*n* = 105)	No. of cases (*n* = 32)	OR (95% CI)^c^
Lead	55	35	1.9 (1.1–3.4)	33	25	1.9 (0.9–3.8)	22	10	2.8 (1.0–7.8)
Mercury	15	7	1.0 (0.4–2.7)	10	5	—	5	2	—
Oil-based paints	38	15	0.7 (0.4–1.4)	26	12	0.7 (0.3–1.6)	12	3	0.7 (0.2–3.1)
Paint thinners	43	22	1.1 (0.6–2.0)	28	15	0.8 (0.4–1.8)	15	7	2.0 (0.7–5.9)
Paint strippers	19	14	1.7 (0.8–3.6)	11	9	1.5 (0.5–4.0)	8	5	2.5 (0.7–9.0)
Varnishes	21	9	0.8 (0.3–2.0)	13	6	—	8	3	—
Adhesive	36	23	1.5 (0.8–2.7)	22	15	1.2 (0.5–2.6)	14	8	2.4 (0.9–6.9)
Dyes or printing inks	37	21	1.4 (0.8–2.7)	22	13	1.1 (0.5–2.4)	15	8	2.5 (0.9–7.2)
Cutting, cooling, or lubricating oils	43	32	1.8 (1.0–3.3)	32	22	1.2 (0.6–2.5)	11	10	6.3 (1.9–20.4)
Gas, diesel fuel, motor or fuel oil	56	29	1.1 (0.6–1.9)	37	22	1.0 (0.5–1.9)	19	7	1.5 (0.5–4.3)
Antifreeze or coolants	23	17	1.6 (0.8–3.3)	17	10	0.9 (0.3–2.4)	6	7	6.3 (1.6–24.1)
Degreasers or cleaning agents	59	28	1.0 (0.6–1.7)	40	20	0.8 (0.4–1.5)	19	8	1.5 (0.5–4.2)
Mineral spirits or white spirits	20	13	1.7 (0.8–3.7)	13	7	1.0 (0.4–2.8)	7	6	4.1 (1.2–14.4)
Solvents (e.g., toluene or xylene)	28	13	1.0 (0.5–2.1)	20	10	0.9 (0.4–2.2)	8	3	1.6 (0.4–7.1)
Dry cleaning agents	4	5	1.9 (0.5–7.8)	3	5	—	1	0	—
Anesthetic gases	6	1	0.4 (0.0–3.2)	4	1	—	2	0	—
Electrical or electronic equipment or machinery	103	53	1.4 (0.9–2.3)	62	37	1.3 (0.7–2.5)	41	16	1.9 (0.8–4.7)
Insecticides	29	15	1.1 (0.5–2.2)	13	11	1.3 (0.5–3.2)	16	4	1.0 (0.3–3.3)
Herbicides	7	4	1.1 (0.3–4.1)	3	2	—	4	2	—
Fungicides	10	1	0.2 (0.0–1.6)	5	1	—	5	0	—
Fumigants	10	5	0.9 (0.3–2.8)	7	4	—	3	1	—

a Individuals with missing information on a specific workplace exposure were excluded from the analysis of that exposure; individuals who did not report a specific exposure served as the reference group for that exposure; for exposure with < 10 cases, stratified analysis was not performed.

b Calculated from logistic regression models; all models were adjusted for age (< 56, 56–65, and > 65 years), sex, area of residence (Boston metropolitan area, eastern Massachusetts, and rest of New England), smoking, and educational level.

c Calculated from logistic regression models; all models were adjusted for age (< 56, 56–65, and > 65 years), sex, area of residence (Boston metropolitan area, eastern Massachusetts, and rest of New England), and educational level.

**Table 4:** Self-reported workplace exposures and the risk of ALS by lifetime days of exposure.^a^

Exposure days	No. of controls (*n* = 253)	No. of cases (*n* = 109)	OR (95% CI)^b^	*p*-Value for trend
Oil-based paints				
1–399	14	7	1.1 (0.4–2.8)	0.33
400–1,999	14	3	0.4 (0.1–1.5)	
≥ 2,000	7	4	0.8 (0.2–3.0)	

Paint thinners				
1–399	14	9	1.5 (0.6–3.8)	0.99
400–1,999	10	3	0.6 (0.2–2.4)	
≥ 2,000	15	8	1.0 (0.4–2.7)	

Paint strippers				
1–399	9	5	1.5 (0.4–4.8)	0.48
400–1,999	3	3	2.5 (0.5–13.3)	
≥ 2,000	6	4	1.1 (0.3–4.2)	

Adhesive				
1–399	10	6	1.4 (0.5–4.2)	0.36
400–1,999	11	7	1.9 (0.7–5.2)	
≥ 2,000	13	8	1.2 (0.4–3.1)	

Dyes or printing inks				
1–399	13	5	1.0 (0.3–2.9)	0.07
400–1,999	16	6	1.0 (0.4–2.6)	
≥ 2,000	6	9	4.0 (1.3–12.1)	

Cutting, cooling, or lubricating oils				
1–399	11	4	0.8 (0.2–2.9)	0.04
400–1,999	7	10	3.6 (1.2–10.5)	
≥ 2,000	23	16	1.8 (0.8–3.8)	

Gas, diesel fuel, motor or fuel oil				
1–399	11	5	1.0 (0.3–3.1)	0.95
400–1,999	14	9	1.5 (0.6–3.7)	
≥ 2,000	29	14	0.9 (0.4–1.9)	

Antifreeze or coolants				
1–399	8	5	1.5 (0.4–5.1)	0.30
400–1,999	4	2	1.0 (0.2–5.6)	
≥ 2,000	11	9	1.7 (0.6–4.5)	

Degreasers or cleaning agents				
1–399	14	7	1.1 (0.4–2.9)	0.92
400–1,999	17	3	0.4 (0.1–1.4)	
≥ 2,000	26	17	1.3 (0.6–2.6)	

Mineral spirits or white spirits				
1–399	6	3	1.8 (0.4–7.7)	0.47
400–1,999	5	5	2.4 (0.6–9.2)	
≥ 2,000	9	4	1.0 (0.3–3.4)	

Solvents (e.g., toluene or xylene)				
1–399	6	2	0.6 (0.1–2.9)	0.39
400–1,999	10	1	0.3 (0.0–2.5)	
≥ 2,000	10	10	2.1 (0.8–5.5)	

Electrical or electronic equipment or machinery				
1–399	8	0	—	0.10
400–1,999	27	16	1.6 (0.8–3.3)	
≥ 2,000	66	36	1.5 (0.8–2.5)	

Insecticides				
1–399	16	9	1.3 (0.5–3.2)	0.84
400–1,999	6	2	0.6 (0.1–3.4)	
≥ 2,000	6	4	1.2 (0.3–4.8)	

a Individuals with missing information on a specific workplace exposure were excluded from the analysis of that exposure; individuals who did not report a specific exposure served as the reference group for that exposure; exposures with < 10 cases were not presented.

b Calculated from logistic regression models; all models were adjusted for age (< 56, 56–65, and > 65 years), sex, area of residence (Boston metropolitan area, eastern Massachusetts, and rest of New England), smoking, and educational level.

**Table 5:** Chemical exposures determined by an industrial hygienist and the risk of ALS, overall and stratified by smoking.^a^

	Overall			Smokers			Nonsmokers		
Exposure	No. of controls (*n* = 253)	No. of cases (*n* =109)	OR (95% CI)^b^	No. of controls (*n* = 148)	No. of cases (*n* = 77)	OR (95% CI)^c^	No. of controls (*n* = 105)	No. of cases (*n* = 32)	OR (95% CI)^c^
Acetone	60	33	1.3 (0.8–2.3)	38	21	0.9 (0.4–1.8)	22	12	2.5 (1.0–6.3)
Aliphatic chlorinated hydrocarbons	67	42	1.6 (0.9–2.7)	46	28	1.0 (0.5–2.0)	21	14	4.1 (1.6–8.9)
Aliphatic hydrocarbons	134	65	1.2 (0.7–1.9)	85	43	0.7 (0.4–1.3)	49	22	2.7 (1.1–6.8)
Aromatic hydrocarbons	111	52	1.1 (0.7–1.8)	69	33	0.7 (0.4–1.3)	42	19	2.5 (1.0–6.2)
Benzene	74	39	1.2 (0.7–2.0)	49	28	0.9 (0.5–1.8)	25	11	2.0 (0.8–5.2)
Benzidine	37	21	1.4 (0.8–2.7)	22	13	1.1 (0.5–2.4)	15	8	2.5 (0.9–7.2)
Carbon tetrachloride	72	33	0.9 (0.6–1.6)	49	24	0.8 (0.4–1.5)	23	9	1.5 (0.6–4.0)
Cyclohexane	87	46	1.2 (0.8–2.1)	57	30	0.8 (0.4–1.6)	30	16	2.7 (1.1–6.6)
Ethyl acetate	57	32	1.3 (0.8–2.3)	37	21	1.0 (0.5–1.9)	20	11	2.6 (1.0–6.8)
Ethylene/propylene glycol	23	17	1.6 (0.8–3.3)	17	10	0.9 (0.3–2.4)	6	7	6.3 (1.6–24.1)
Glycol ethers	53	33	1.6 (0.9–2.7)	35	19	0.9 (0.4–1.8)	18	14	5.1 (1.9–13.7)
Heptane	36	23	1.5 (0.8–2.7)	22	15	1.2 (0.5–2.6)	14	8	2.4 (0.9–6.9)
*n*-Hexane	44	29	1.7 (1.0–3.0)	28	18	1.2 (0.6–2.4)	16	11	3.4 (1.3–9.6)
Methanol	47	24	1.1 (0.6–2.0)	30	16	0.8 (0.4–1.7)	17	8	1.9 (0.7–5.2)
Methyl chloroform	59	28	1.0 (0.6–1.7)	40	20	0.8 (0.4–1.5)	19	8	1.5 (0.5–4.2)
Methyl ethyl ketone	57	32	1.3 (0.8–2.3)	37	21	1.0 (0.5–1.9)	20	11	2.6 (1.0–6.8)
Methylene chloride	69	33	1.0 (0.6–1.7)	45	22	0.7 (0.4–1.4)	24	11	1.8 (0.7–4.6)
Methyl *tert*-butyl ether	56	29	1.1 (0.6–1.9)	37	22	1.0 (0.5–1.9)	19	7	1.5 (0.5–4.3)
Naphtha (VM&P)	92	46	1.1 (0.7–1.8)	58	33	0.9 (0.5–1.8)	34	13	1.4 (0.6–3.5)
Perchloroethylene	72	35	1.0 (0.6–1.7)	49	26	0.8 (0.4–1.6)	23	9	1.5 (0.6–4.0)
Stoddard solvent	68	35	1.1 (0.7–1.9)	44	25	0.9 (0.4–1.7)	23	10	1.7 (0.7–4.3)
Tetraethyl lead	56	29	1.1 (0.6–1.9)	37	22	1.0 (0.5–1.9)	19	7	1.5 (0.5–4.3)
Toluene	114	55	1.1 (0.7–1.8)	71	36	0.8 (0.4–1.4)	43	19	2.4 (1.0–5.9)
Trichloroethylene	72	35	1.0 (0.6–1.7)	49	26	0.8 (0.4–1.6)	23	9	1.5 (0.6–4.6)
Trichlorotrifluoroethane	59	28	1.0 (0.6–1.7)	40	20	0.8 (0.4–1.5)	19	8	1.5 (0.5–4.2)
Xylene	77	37	1.2 (0.7–1.9)	49	22	0.7 (0.4–1.4)	28	15	3.1 (1.2–8.1)

VM&P, varnish makers and painters.

a Individuals with missing information on a specific chemical were excluded from the analysis of that chemical; individuals never exposed to the chemical in the question served as the reference group for that chemical.

b Calculated from logistic regression models; all models were adjusted for age (< 56, 56–65, and > 65 years), sex, area of residence (Boston metropolitan area, eastern Massachusetts, and rest of New England), smoking, and educational level.

c Calculated from logistic regression models; all models were adjusted for age (< 56, 56–65, and > 65 years), sex, area of residence (Boston metropolitan area, eastern Massachusetts, and rest of New England), and educational level.

**Table 6:** Workplace formaldehyde exposure and the risk of ALS.^a^

	No. of controls (*n* = 253)	No. of cases (*n* = 109)	OR (95% CI)^b^
Never	204	89	Ref.
Ever	49	20	0.8 (0.5–1.5)
Exposure probability^c^			
0–1	7	2	0.6 (0.1–2.8)
1	27	9	0.7 (0.3–1.6)
2	15	9	1.3 (0.5–3.2)
*p*-Value for trend			0.50
Weighted exposure duration^d^			
≤ 10,000 hr	14	7	1.1 (0.4–2.8)
10,001–40,000 hr	19	8	0.8 (0.3–1.9)
> 40,000 hr	16	5	0.7 (0.2–2.0)
*p*-Value for trend			0.45

a Individuals who never had a formaldehyde-related occupation served as the reference group.

b Calculated from logistic regression models; all models were adjusted for age (< 56, 56–65, and > 65 years), sex, area of residence (Boston metropolitan area, eastern Massachusetts, and rest of New England), smoking, and educational level.

c Highest probability of exposure ever experienced: three-level scale in 0–1, 1, and 2.

d Level 0–1 was given a weight of 0.5; level 1 was given a weight of 1; and level 2 was given a weight of 2.
